# Cerebrospinal Fluid and Microdialysis Cytokines in Severe Traumatic Brain Injury: A Scoping Systematic Review

**DOI:** 10.3389/fneur.2017.00331

**Published:** 2017-07-10

**Authors:** Frederick A. Zeiler, Eric Peter Thelin, Marek Czosnyka, Peter J. Hutchinson, David K. Menon, Adel Helmy

**Affiliations:** ^1^Department of Surgery, Section of Neurosurgery, University of Manitoba, Winnipeg, MB, Canada; ^2^Clinician Investigator Program, University of Manitoba, Winnipeg, MB, Canada; ^3^Department of Anesthesia, Addenbrooke’s Hospital, University of Cambridge, Cambridge, United Kingdom; ^4^Division of Neurosurgery, Department of Clinical Neurosciences, University of Cambridge, Cambridge Biomedical Campus, Cambridge, United Kingdom; ^5^Department of Clinical Neuroscience, Karolinska Institute, Stockholm, Sweden

**Keywords:** cytokines, traumatic brain injury, brain injury, systematic review, microdialysis, cerebrospinal fluid

## Abstract

**Objective:**

To perform two scoping systematic reviews of the literature on cytokine measurement in: 1. cerebral microdialysis (CMD) and 2. cerebrospinal fluid (CSF) in severe traumatic brain injury (TBI) patients.

**Methods:**

Two separate systematic reviews were conducted: one for CMD cytokines and the second for CSF cytokines. Both were conducted in severe TBI (sTBI) patients only.

**Data sources:**

Articles from MEDLINE, BIOSIS, EMBASE, Global Health, Scopus, Cochrane Library (inception to October 2016), reference lists of relevant articles, and gray literature were searched.

**Study selection:**

Two reviewers independently identified all manuscripts utilizing predefined inclusion/exclusion criteria. A two-tier filter of references was conducted.

**Data extraction:**

Patient demographic and study data were extracted to tables.

**Results:**

There were 19 studies identified describing the analysis of cytokines *via* CMD in 267 sTBI patients. Similarly, there were 32 studies identified describing the analysis of CSF cytokines in 1,363 sTBI patients. The two systematic reviews demonstrated: 1. limited literature available on CMD cytokine measurement in sTBI, with some preliminary data supporting feasibility of measurement and associations between cytokines and patient outcome. 2. Various CSF measured cytokines may be associated with patient outcome at 6–12 months, including interleukin (IL)-1b, IL-1ra, IL-6, IL-8, IL-10, and tumor necrosis factor 3. There is little to no literature in support of an association between CSF cytokines and neurophysiologic or tissue outcomes.

**Conclusion:**

The evaluation of CMD and CSF cytokines is an emerging area of the literature in sTBI. Further, large prospective multicenter studies on cytokines in CMD and CSF need to be conducted.

## Introduction

Neuroinflammation after traumatic brain injury (TBI) is postulated to be a key driver of secondary brain injury in the acute/subacute phase after injury (1, 2). Upregulation of various components of the inflammatory cascade have been associated with lesion expansion (3), cerebral edema (4), derangements in neural transmission (5), and subsequent tissue death (6) in animal models of stroke and TBI. In humans, the inflammatory process post-TBI has been of interest, since its therapeutic modulation can potentially lead to amelioration of pathophysiology, tissue salvage, and improved patient outcomes (7, 8). Serum cytokine levels are easily measured in TBI patients, and elevation in pro-inflammatory cytokines have been associated with worse patient outcome (9, 10). However, systemic cytokine levels can be confounded by extracranial pathology and variable blood–brain barrier leak of centrally derived mediators. Measurement of cerebral levels of cytokines provides a more direct metric of neuroinflammation following TBI, but, to date, the measurement of cerebral microdialysis (CMD) (11–29) and cerebrospinal fluid (CSF) (30–65) cytokines have been limited to small studies.

The goal of this study was to produce a scoping systematic review of the literature on both CMD and CSF cytokines in severe TBI (sTBI). Our hope was to produce a comprehensive overview of the literature on this emerging topic.

## Methods

Two separate scoping systematic reviews were conducted, using the methodology outlined in the Cochrane Handbook for Systematic Reviewers (66). Data were reported following the preferred reporting items for systematic reviews and meta-analyses (67). The review questions and search strategy were decided upon by the primary author (Frederick A. Zeiler) and supervisors (Adel Helmy and David K. Menon).

This manuscript was conducted in concert with a similar review on cytokines in CMD and CSF for aneurysmal subarachnoid hemorrhage (SAH) patients.

### Search Question and Population of Interest

Given that two separate systematic reviews were conducted, one for CMD cytokines and the other for CSF cytokines, two distinct questions were posed. The limited literature on CMD cytokines identified through a preliminary search of PubMed led us to conduct a scoping review for the CMD cytokine search. We attempted to identify all studies in this area to date, and all articles describing microdialysis cytokine measures in humans with sTBI included in our review in order to provide a comprehensive overview of this emerging area of literature. The key question for this part of the review was:

- What literature has been published on CMD of cytokines in sTBI?

The larger literature base for CSF cytokines in TBI led us to narrow our question for this scoping review, focusing on relevant outcomes (see below). The questions posed for this scoping systematic review was:

- Is there literature to suggest an association between CSF cytokine measures in sTBI and patient outcome, neurophysiologic outcome, or tissue outcome?

For the CSF cytokine review, the primary outcome measures were documented association between CSF cytokine levels and: patient outcome, neurophysiologic outcome (as measured *via* intensive care unit (ICU)-based monitoring; intracranial pressure (ICP)/cerebral perfusion pressure (CPP), brain tissue oxygen monitoring (PbtO_2_), thermal diffusion assessment of cerebral blood flow (CBF), transcranial Doppler (TCD) measure of cerebral blood flow velocity (CBFV), any neuroimaging based assessment of CBF/perfusion, and electrophysiology), and tissue outcome [as assessed on follow-up neuroimaging by either computed tomography (CT) or magnetic resonance imaging]. Any outcome score or mention of morbidity/mortality within the studies was deemed acceptable for documentation of patient outcome. Secondary outcome measures were complications associated with CSF monitoring of cytokines.

The list of included cytokines in CMD or CSF included: interleukin (IL)-1a, IL-1b, IL-1ra, IL-2, sIL-2ra, IL-3, IL-4, IL-5, IL-6, IL-7, IL-8, IL-9, IL-10, IL-11, IL-12, IL-12p70, IL-13, IL-14, IL-15, IL-16, IL-17, inducible protein (IP)-10, eotaxin, tumor necrosis factor (TNF), interferon gamma (INF-g), monocyte chemoattractant proteins, macrophage inflammatory proteins (MIPs), transforming growth factor (TGF), nerve growth factor (NGF), brain-derived neurotrophic factor, glial-derived neurotrophic factor, soluble tumor necrosis factor receptor (sTNFR), granulocyte macrophage colony stimulating factor, soluble FAS, soluble vascular cell adhesion molecule (sVCAM)-1, and soluble intracellular adhesion molecule (sICAM)-1, platelet-derived growth factor, regulated on activation, normal T cell expressed and secreted (RANTES), macrophage-derived chemokine (MDC), fms-like tyrosine kinase 3 (Flt3), Fractalkine, and fibroblast growth factor receptor.

### Inclusion/Exclusion Criteria

#### CMD Cytokine Review

Inclusion criteria were: all studies including human subjects with sTBI (GCS 8 or less), any study size, any age category, CMD analysis for cytokines, and mention of any outcome (patient based or otherwise). Exclusion criteria were: non-English studies and animal studies.

#### CSF Cytokine Review

Inclusion criteria were: all studies including human subjects with sTBI (GCS of 8 or less), studies with 10 or more patients, any age category, CSF analysis for cytokines, and documentation either: patient functional outcome, neurophysiologic outcome, or tissue outcome in relation to CSF cytokine measures. Exclusion criteria were: non-English studies, animal studies, and studies of less than 10 patients. Non-English studies were excluded given the small number identified.

### Search Strategies

MEDLINE, BIOSIS, EMBASE, Global Health, SCOPUS, and Cochrane Library from inception to October 2016 were searched using individualized search strategies. The search strategy for the CMD scoping systematic review using MEDLINE can be seen in Appendix A in Supplementary Material, with a similar search strategy utilized for the other databases. Further, the search strategy for the CSF scoping systematic review using MEDLINE can be seen in Appendix B in Supplementary Material, with similar strategies employed for the other databases.

In addition, we surveyed relevant meeting proceedings for the last 5 years looking for ongoing and unpublished work based on cytokine analysis *via* CMD or CSF in sTBI patients. The meeting proceedings of the following professional societies were searched: Canadian Neurological Sciences Federation, American Association of Neurological Surgeons, Congress of Neurological Surgeons, European Neurosurgical Society, World Federation of Neurological Surgeons, National Neurotrauma Society, American Neurology Association, American Academy of Neurology, European Federation of Neurological Science, World Congress of Neurology, Society of Critical Care Medicine, Neurocritical Care Society, European Society for Intensive Care Medicine, World Federation of Societies of Intensive and Critical Care Medicine, American Society for Anesthesiologists, World Federation of Societies of Anesthesiologist, Australian Society of Anesthesiologists, International Anesthesia Research Society, Society of Neurosurgical Anesthesiology and Critical Care, Society for Neuroscience in Anesthesiology and Critical Care, Japanese Society of Neuroanesthesia and Critical Care, International NeuroTrauma Society, International Brain Injury Association, and the College of Intensive Care Medicine Annual Scientific Meeting (CICMASM—Australia).

Finally, reference lists of any review articles on CSF or CMD cytokines were reviewed for any missed relevant studies.

### Study Selection

Utilizing two reviewers, a two-step review of all articles returned by our search strategies was performed. First, the reviewers independently (Frederick A. Zeiler and Eric Peter Thelin) screened titles and abstracts of the returned articles to decide if they met the inclusion criteria. Second, full text of the chosen articles was then assessed to confirm if they met the inclusion criteria and that the primary outcomes of interest were reported in the study (Frederick A. Zeiler and Eric Peter Thelin). Any discrepancies between the two reviewers were resolved by a third reviewer if needed (Adel Helmy or David K. Menon).

### Data Collection

Data were extracted from the selected articles and stored in an electronic database. Data fields included: patient demographics, type of study, article location, number of patients, CMD/CSF substrate measured, CMD/CSF measurement details (probe tissue location, sampling frequency), outcome measure described (patient, neurophysiologic, tissue), association between CMD/CSF cytokine measure to outcome, and complications. All extracted data can be found in Tables 1 through 4, with study designs in Tables 1 and 2, and study outcomes in Tables 3 and 4.

**Table 1:** CMD cytokine study characteristics and patient demographics.

Reference	Number of patients	Study type	Article location	Mean age (years)	Patient characteristics	Primary and secondary goal of study
Cederberg et al. (11)	7	Retrospective case series	Meeting abstract	Unknown “children”	Severe TBI; 3 underwent DC	Primary: to compare CMD cytokines to common CMD measures, PbtO_2_, and ICP
						Secondary: none mentioned

Figaji et al. (12)	5	Unknown	Meeting abstract	Unknown “children”	Severe TBI	Primary: to compare CMD cytokine and other CMD measures
						Secondary: none mentioned

Guilfoyle et al. (13)	12	Prospective observational	Meeting abstract	Unknown “adults”	Severe TBI	Primary: to compared CMD cytokine measures in healthy vs. peri-lesional tissue
						Secondary: none mentioned

^a^Helmy et al. (14)	12	Prospective observational	Manuscript	Unknown “adults”	Severe TBI	Primary: to perform a principle component analysis of CMD cytokines to determine cytokine patterns and temporal profiles
						Secondary: none mentioned

^a^Helmy et al. (15)	12	Prospective observational	Manuscript	Unknown “adults”	Severe TBI	Primary: 1. To compare crystalloid vs. albumin perfusate in CMD cytokine recovery. 2. To compare the cytokine profile in sTBI
						Secondary: not specified

^b^Helmy et al. (16)	20	Prospective RCT	Manuscript	38.9 years (range: 18–61 years)	Severe diffuse TBI; randomized to subcutaneous rhIL-1ra	Primary: 1. To provide safety data in a randomized fashion on rhIL-1ra in sTBI
						2. To describe the impact of rhIL-1ra on CMD cytokine profiles
						Secondary: none mentioned

^b^Helmy et al. (17)	20	Retrospective database analysis	Manuscript	38.9 years (range: 18–61 years)	Severe diffuse TBI; randomized to subcutaneous rhIL-1ra	Primary: to retrospectively analyze RCT data on rhIL-1ra administration, to better delineate the temporal change in cytokine profiles
						Secondary: none mentioned

Hillman et al. (18)	9 (10 total, but failed CMD catheter in 1)	Prospective observational	Manuscript	Unknown	Severe brain injury (undisclosed number of aSAH and sTBI patients)	Primary: to evaluate newer microdialysis catheters and their ability to measure various CMD macromolecules (including IL-6) vs. older catheters. Varied perfusates were also analyzed
						Secondary: none mentioned

Hillman et al. (19)	7 with TBI (14 total; mixed injury sources)	Prospective observational	Manuscript	Unknown	sTBI—5 requiring “surgery”	Primary: to determine the CMD cytokine patterns in TBI
						Secondary: none mentioned

Hutchinson et al. (20)	15	Prospective observational	Manuscript	41 years (range: 17–68 years)	Severe TBI	Primary: to determine the feasibility of measures IL-1a, IL-1b, and IL-1ra in CMD samples
						Secondary: correlation of cytokine to ICP, CPP, and patient outcome

Mellergard et al. (21)	7 (total 38 patients; only 7 with TBI)	Prospective observational	Manuscript	Unknown	Severe TBI	Primary: to evaluate CMD cytokine profiles immediately after insertion of the CMD catheter
						Secondary: none mentioned

^c^Mellergard et al. (22)	57 (total 145 patients; only 57 with TBI)	Retrospective case series	Manuscript	Unknown	Severe TBI	Primary: to determine the CMD cytokine responds to TBI
						Secondary: none mentioned

^c^Mellergard et al. (23)	57 (total 145 patients; only 57 with TBI)	Retrospective case series	Manuscript	Unknown	Severe TBI	Primary: to determine the CMD cytokine responds to TBI
						Secondary: none mentioned

Mellergard et al. (24)	69	Unknown	Manuscript	45.9 years (range: unknown)	Severe TBI	Primary: to determine if there is age-related difference in CMD cytokines
						Secondary: none mentioned

Mondello et al. (25)	6	Prospective observational	Meeting abstract	Unknown	Severe TBI	Primary: to evaluate the temporal profile of CMD and CSF cytokines in TBI
						Secondary: none mentioned

Parez-Barcena et al. (26)	16	Prospective observational	Manuscript	31.8 years (range: 16–65 years)	Severe diffuse TBI	Primary: to determine the cytokine profiles in severe diffuse TBI patients
						Secondary: to determine the correlation between cytokines and ICP, PbtO_2_, and CT changes

Roberts et al. (27)	8	Prospective observational	Manuscript	43.4 years (range: unknown)	Severe TBI	Primary: to measure the blood/CSF/CMD MMP and cytokine response post-TBI
						Secondary: correlation to neurologic exam, ICP, PbtO_2_, GOS at discharge

Winter et al. (28)	3	Prospective observational	Manuscript	Unknown	Severe TBI	Primary: to describe the technique of cytokine measurement *via* CMD
						Secondary: describe cytokine patterns in TBI

Winter et al. (29)	14	Prospective observational	Manuscript	43.1 years (range: 21–77 years)	Severe TBI	Primary: to evaluate the changes in CMD cytokines post-TBI
						Secondary: correlation to patient outcome

*TBI, traumatic brain injury; sTBI, severe TBI; aSAH, aneurysmal subarachnoid hemorrhage; DC, decompressive craniectomy; CMD, cerebral microdialysis; RCT, randomized control trial; ICP, intracranial pressure; CPP, cerebral perfusion pressure; CSF, cerebrospinal fluid; LPR, lactate:pyruvate ratio; CT, computed tomography; PbtO_2_, partial pressure of oxygen in brain tissue; MMPs, matrix metalloproteins, IL, interleukin; a, alpha; b, beta; ra, receptor anatogonist; rh, recombinant human*.

*^a^Same patient population reported in both Helmy et al. (14) and Helmy et al. (15)*.

*^b^Same patient population described in Helmy et al. (16) and Helmy et al. (17)*.

*^c^Same patient population reported in both Mellergard et al. (22) and Mellergard et al. (23)*.

**Table 2:** CSF cytokine study characteristics and patient demographics.

Reference	Number of patients	Study type	Article location	Mean age (years)	Patient characteristics	Primary and secondary goal of study
**Patient functional outcome**						
Abboud et al. (30)	31	Prospective observational	Manuscript	31.6 years (range: unknown)	Severe TBI	Primary: to describe the correlation between CSF cytokine profiles and outcome at 6 and 12 months
						Secondary: none mentioned

Bell et al. (31)	15	Prospective observational	Manuscript	6.1 years (range: 0.1–16 years)	Severe TBI	Primary: to determine the relationship between IL-6 and IL-10 with patient outcome
						Secondary: to compare CSF cytokine levels to non-TBI control subjects (*n* = 20)

Chiaretti et al. (32)	29	Prospective observational	Manuscript	9.7 years (range: 1.3–15.6 years)	Severe TBI	Primary: to determine the association between IL-6 and patient outcome
						Secondary: to determine the correlation between IL-6 and NGF in CSF. Also to compare to non-TBI control patients (*n* = 31)

Chiaretti et al. (33)	27	Prospective observational	Manuscript	8.6 years (range: 1.3–15.6)	Severe TBI	Primary: to determine the association between IL-1b, IL-6, NGF, BDNF, and GDNF with patient outcome
						Secondary: none mentioned

Chiaretti et al. (34)	14	Prospective observational	Manuscript	7.8 years (range: 0.3–15.6 years)	Severe TBI	Primary: to determine the relationship between IL-1b and IL-6 with patient outcome
						Secondary: to compared cytokine expression to obstructive hydrocephalus controls

Hans et al. (35)	11	Prospective observational	Manuscript	36.7 years (range: 16–67)	Severe TBI	Primary: to determine the association between IL-6 and sIL-6R to patient outcome
						Secondary: to compare these CSF cytokine levels to those in plasma

Hayakata et al. (36)	53	Prospective observational	Manuscript	34–49 years	Severe TBI	Primary: to determine the association between TNF-a, IL-1, IL-6, IL-8, and IL-10 with patient outcome
						Secondary: to determine the association between cytokines and S100B expression in CSF. Also compare cytokines to ICP

Jamil et al. (37)	61	Prospective observational	Meeting abstract	Unknown “adults”	Severe TBI	Primary: to determine the relationship between acute measures of CSF cytokines and PTD at 6 and 12 months
						Secondary: none mentioned

Juengst et al. (38)	25	Prospective observational	Meeting abstract	Unknown “adults”	Severe TBI	Primary: to determine the association between acute cytokine levels and apathy at 6 and 12 months post-injury
						Secondary: none mentioned

Juengst et al. (39)	37	Prospective observational	Manuscript	“Adults”	Moderate–severe TBI	Primary: to determine the relationship between TNF-a and disinhibition/suicidality post-TBI
				Unclear overall mean age		Secondary: compare levels in CSF and serum to healthy controls (*n* = 15)

Juengst et al. (40)	50	Prospective observational	Manuscript	31.3 years (range: unknown)	Severe TBI	Primary: to determine the relationship between acute CSF cytokine profiles and the risk of PTD at 6 and 12 months post-injury
						Secondary: none mentioned

Kirchhoff et al. (41)	23	Prospective observational	Meeting abstract	Unknown	Severe TBI	Primary: to determine the IL-10 response in CSF in TBI patients. Also determine the relationship to outcome.
						Secondary: compared CSF in TBI to elective surgical patients (*n* = 10)

Kossmann et al. (42)	22	Prospective observational	Manuscript	41 years (range: 17–73)	Severe TBI	Primary: to determine the relationship between CSF IL-6 and NGF. Also determine the association to patient outcome.
						Secondary: compare IL-6 and NGF in controls (*n* = 3)

Kumar et al. (43)	114	Prospective observational	Manuscript	Unclear overall mean age	Severe TBI	Primary: to determine the relationship of IL-6 in CSF to serum values and patient outcome
						Secondary: compare CSF levels in non-TBI controls (*n* = 23)

Kumar et al. (44)	111	Prospective observational	Manuscript	Unknown (range: 16–75)	Severe TBI	Primary: to utilize PCA to determine clusters of cytokines associated with patient outcome
						Secondary: to determine a temporal patter of cytokine clusters and relationship to outcome

Kushi et al. (45)	22	Prospective observational	Manuscript	45 years (range: unknown)	Severe TBI	Primary: to compare CSF and Serum IL-6/IL-8 levels and determine the association to patient outcome
						Secondary: none mentioned

Nwachuku et al. (46)	32	Prospective observational	Manuscript	31 years (range: unknown)	Severe TBI	Primary: to determine the association between various CSF cytokines and patient outcome
						Secondary: none mentioned

Santarseiri et al. (47)	91	Prospective observational	Manuscript	35.8 years (range: 16–73)	Severe TBI	Primary: to identify CSF cytokines associated with patient outcome. Also determine association between cytokines and neuroendocrine cortisol function
						Secondary: none mentioned

Shiozaki et al. (48)	35	Prospective observational	Manuscript	39 years (range: 14–77 years)	Severe TBI	Primary: to determine the association between CSF cytokine profiles and patient outcome
						Secondary: to determine the association between cytokines and ICP

Singhal et al. (49)	36	Prospective observational	Manuscript	34.4 years (range: 17–68 years)	Severe TBI	Primary: to determine the association between cytokines and electrophysiologic/functional patient outcome
						Secondary: none mentioned

Whalen et al. (50)	27	Prospective observational	Manuscript	Unknown “children”	Severe TBI	Primary: to determine the association between CSF IL-8 levels and patient outcome
						Secondary: to determine the association between CSF IL-8 in TBI patients and non-TBI controls (*n* = 24)

**Neurophysiologic association**						

Muller et al. (51)	25	Prospective observational	Manuscript	41 years (range: unknown)	Severe TBI	Primary: to evaluate the relationship between CSF IL-6, IL-8, and IL-10 with TCD defined CBF
						Secondary: none mentioned

Stein et al. (52)	14 with CSF cytokines	Prospective observational	Manuscript	31.6 years (range: unknown)	Severe TBI	Primary: to determine the relationship between CSF cytokines with ICP and patient outcome
						Secondary: none mentioned

**Nil association studies**						

Amick et al. (53)	24	Prospective observational	Manuscript	5.4 years (range: 0.2–16 years)	Moderate–severe TBI	Primary: to determine the association between IL-2, IL-4, IL-6 and IL-12 with patient outcome
						Secondary: compare IL levels in CSF to non-TBI controls (*n* = 12)

Buttram et al. (54)	36	Prospective observational	Manuscript	6.9 years (range: unknown)	Severe TBI	Primary: to measure CSF cytokines and determine the impact of moderate hypothermia on expression. Also determine the link between CSF cytokines and outcome
						Secondary: compared CSF cytokine profile to non-TBI controls (*n* = 10)

Csuka et al. (55)	28	Prospective observational	Manuscript	36 years (range: 16–67 years)	Severe TBI	Primary: to determine the association between various CSF and serum cytokines
						Secondary: to determine the association between CSF cytokines with outcome and ICP

Diamond et al. (56)	59 with CSF cytokines	Prospective observational	Manuscript	Unclear mean age for CSF cytokine cohort	Moderate–severe TBI	Primary: to determine the association between serum and CSF cytokine levels with the development of PTE
						Secondary: to compare serum and CSF levels with healthy control values. Also assess genetic IL-1b associations with PTE

Goodman et al. (57)	23	Prospective observational	Manuscript	32.7 years (range: 15–57 years)	Severe TBI	Primary: to compare CSF and jugular venous cytokine profiles
						Secondary: to compare cytokine profiles to ICP and CPP

Gopcevic et al. (58)	20	Prospective observational	Manuscript	53 years (range: unknown)	Severe TBI	Primary: to determine the association between jugular serum and CSF IL-8 levels with in-hospital mortality
						Secondary: to determine the association between jugular plasm and CSF IL-8 levels

Lenzlinger et al. (59)	41	Prospective observational	Manuscript	38 years (range: 15–74 years)	Severe TBI	Primary: to determine the association between CSF and serum cytokines with patient outcome
						Secondary: to compared serum and CSF cytokine profiles

Maier et al. (60)	29	Prospective observational	Manuscript	54.8 years (range: 16–85 years)	Severe TBI	Primary: to determine the CSF profile for two soluble tumor necrosis factor receptors (TNFR’s)
						Secondary: to determine the association between CSF sTNFR levels and patient outcome

Maier et al. (61)	29	Prospective observational	Manuscript	45.5 years (range: 18–75 years)	Severe TBI	Primary: to evaluate the correlation between CSF and serum cytokine
						Secondary: to determine the association between cytokine profile and patient outcome. Also, compare to CSF from healthy volunteers (*n* = 35)

Morganti-Kossmann et al. (62)	42	Unclear	Meeting abstract	Unknown	Severe TBI with various primary and secondary injuries	Primary: to determine the association between serum and CSF cytokines with injury patterns
						Secondary: to determine the association between cytokine profiles and patient outcome

Newell et al. (63)	66	Retrospective case series	Manuscript	6 years (range: 0.1–16 years)	Severe TBI	Primary: to measure inflammatory markers in the CSF linked to T-cell activation
						Secondary: to comment on the association between these markers and patient outcome. Also compare levels to healthy controls

Ross et al. (64)	50	Prospective observational	Manuscript	21 years (range: 4–70 years)	Severe TBI	Primary: to compare serum and CSF TNF-a in TBI patients to healthy controls (*n* = 46)
						Secondary: to compare TNF-a levels to patient outcome

Uzan et al. (65)	11	Prospective observational	Manuscript	28.5 years (range: 2.5–53 years)	Severe TBI	Primary: to determine the association between NO metabolic products and IL-8
						Secondary: to determine the association between NO and IL-8 with patient outcome

*TBI, traumatic brain injury; sTBI, severe TBI; RCT, randomized control trial; ICP, intracranial pressure; CPP, cerebral perfusion pressure; CSF, cerebrospinal fluid; CBF, cerebral blood flow; PbtO_2_, partial pressure of oxygen in brain tissue; TCD, transcranial Doppler; DC, decompressive craniectomy; IL, interleukin; a, alpha; b, beta; ra, receptor antagonist; TNF, tumor necrosis factor; NO, nitrous oxide; TNFR, tumor necrosis factor receptor; NGF, nerve growth factor; BDNF, brain-derived neurotrophic factor; GDNF, glial-derived neurotrophic factor; PTE, post-traumatic epilepsy; PTD, post-traumatic depression; PCA, principle component analysis*.

**Table 3:** CMD cytokine measures and outcomes.

Reference	Catheter location and measured CMD cytokines	Interventional therapies applied during measurement	Primary outcome	Secondary outcome	Complications to CMD	Conclusions
Cederberg et al. (11)	Mixed locations	Not specified	6/7 patients survived	N/A	Not specified	IL-6/IL-8 are increase in CDM both in “healthy” and peri-lesional tissue
	IL-6/IL-8		IL-6 and IL-8 was increased in survivors			
	Unclear sampling interval [3 samples in each patient over course of intensive care unit (ICU) stay]		Peri-lesional location of CMD catheter yielded higher IL-6 and IL-8 levels			
	Perfusate not specified					

Figaji et al. (12)	Unclear locations	Not specified	Variable individual cytokine responses	N/A	Not specified	IL-6/IL-8 are consistently increased in CMD in pediatric sTBI
	IL-1a, IL-1b, IL1-ra, IL-6, IL-8, and IL-10; VEGF, and MCP-1		IL-6 and IL-8 were the most consistently elevated across all patients			
	Unclear sampling interval					
	Perfusate not specified					

Guilfoyle et al. (13)	2× CMD catheters per patients (1 healthy tissue, 1 peri-lesional)	Not specified	IL-7 (*p* < 0.05) and IL-8 (<0.05) were found to be higher in peri-lesional tissue IL-1b and interferon gamma (INF-g) were higher in peri-lesional tissue within the first 72 h post-injury	N/A	Not specified	IL-7/IL-8 are higher in peri-lesional tissue IL-1b and INF-g are higher in peri-lesional tissue within the first 72 h
	“42 cytokines” IL-7 and IL-8					
	Unclear sampling interval					
	Perfusate not specified					

^a^Helmy et al. (14)	Area of “diffuse injury”	Not specified	IL-1b and TNF are covariate MIP-1a and MIP-1b were coexpressed Earlier temporal expression of IL-6, GRO, G-CSF, IP10 compared to IL-10, MCP-3, IL-17	N/A	Not specified	PCA of CMD cytokine profiles yields covariate relationships between specific cytokines and temporal expression pattern
	EGF, Eotaxin, FGF-2, fms-like tyrosine kinase 3 (Flt3) lig, Frac, G-CSF, GM-CSF, GRO, IFN-a2, IFN-g, IL-1a, IL-1b, IL-1ra, IL-2, IL-3, IL-4, IL-5, IL-6, IL-7, IL-8, IL-9, IL-10, IL-12p40, IL-12p70, IL-13, IL-15, IL-17, inducible protein (IP)-10, MCP-1, MCP-3, MDC, MIP-1a, MIP-1b, PDGF-AA, PDGF-AAAB, regulated on activation, normal T cell expressed and secreted (RANTES), sCD40L, sIL-2R, TGF-a, TNF		IL-1ra and IL-1a are covariate MIP-1a and MIP-1b were coexpressed Earlier temporal expression of IL-6, GRO, G-CSF, IP10 compared to IL-10, MCP-3, IL-17			
	q6 h pooled sampling over 5 days					
	3.5% human ablumin solution perfusate					

^a^Helmy et al. (15)	Double side-by-side in six patients (to analyze perfusate), and single catheter in six patients—unclear tissue location	Unclear; two patients under went DC for refractory ICP	Albumin perfusate led to significantly higher fluid recovery compared to crystalloid. Albumin perfusate led to significantly higher cytokine recovery (18 cyotkines) Brain concentrations of 23 cytokines were significantly higher than jugular plasma concentrations (ex. IL-1ra, IL-1a, IL-1b, IL-6, IL-8, IL-10, IL-12p70, MCP-1) Many cytokines displayed a temporal expression, with expression within the first 72 h (e.g., TNF, IL-7, IL-8, MIP1a, sCD40L, IL-1β, GRO, PDGF, AA, RANTES, MIP-1b, IL-1ra, G-CSF, IP10, IL-6)	N/A	Not specified	Ablumin CMD perfusate led to increased fluid and cytokine recovery Brain cytokine concentrations were significantly higher than jugular plasma for 23 cytokines. Many cytokines displayed a temporal expression patter with early expression post-injury (72 h)
	EGF, Eotaxin, FGF-2, Flt3 lig, Frac, G-CSF, GM-CSF, GRO, IFN-a2, IFN-g, IL-1a, IL-1b, IL-1ra, IL-2, IL-3, IL-4, IL-5, IL-6, IL-7, IL-8, IL-9, IL-10, IL-12p40, IL-12p70, IL-13, IL-15, IL-17, IP-10, MCP-1, MCP-3, MDC, MIP-1a, MIP-1b, PDGF-AA, PDGF-AAAB, RANTES, sCD40L, sIL2R, TGFa, TNF					
	q6 h pooled sampling over 5 days					
	Assessed both crystalloid and 3.5% human albumin perfusate					

^b^Helmy et al. (16)	Right frontal location (in setting of diffuse injury) 42 cytokine array q6 h pooled sampling for 5 days Isotonic central nervous system perfusate	Group 1 (*n* = 10): after baseline 6 h CMD samples; received 100 mg rhIL-1ra subcut	No complications secondary to rhIL-1ra were seen. CMD IL-1ra concentrations were significantly higher in the treatment group vs. control (*p* = 0.02), with variation over time (*p* < 0.0001) MDC was significantly lower in the rhIL-1ra (*p* = 0.05)	N/A	Not specified	rhIL-1ra appears safe in severe diffuse TBI rhIL-1ra reaches the brain extracellular fluid MDC was lower in the rhIL-1ra group
		Repeated q24 h for total of five doses				
		Group 2 (*n* = 10): control group				
		No specifics on other ICU therapies				

^b^Helmy et al. (17)	Right frontal location (in setting of diffuse injury) 42 cytokine array q6 h pooled sampling for 5 days 3.5% human albumin perfusate	Group 1 (*n* = 10): after baseline 6 h CMD samples; received 100 mg rhIL-1ra subcut	Based on PCA it was found that cytokines associated with macrophage recruitment were decreased in the rhIL-1ra group (MIP-1a, MCP-3, Fractalkine, GM-CSF)	N/A	Not specified	CMD macrophage base cytokines are decreased in rhIL-1ra-treated patients
		Repeated q24 h for total of five doses				
		Group 2 (*n* = 10): control group				
		No specifics on other ICU therapies				

Hillman et al. (18)	Paired CMD catheter placement in peri-lesional tissue	Not specified	CMD IL-6 concentrations varied depending on underlying condition and secondary injury (i.e., ischemia)	N/A	1 catheter membrane failure	CMD IL-6 concentrations varied from patient to patient and depending on initial and secondary injury patterns
	IL-6					
	q6 h pooled analysis		The temporal expression of CMD measured IL-6 varied between patients			
	Ringer’s/dextran 60 or human albumin perfusate					

Hillman et al. (19)	Peri-lesional tissue IL-1b, IL-6 q6 h pooled analysis 3.5% human albumin perfusate	Not specified	CMD biochemical evidence of ischemia (LPR > 30 and glutamate >80 μmol/L for 24 h period) was associated with significant IL-6 increase (*p* < 0.01), which subsided after ~90 h post-injury (*p* < 0.001)	N/A	Not specified	CMD IL-6 displays a correlation with CMD biochemical ischemia and a temporal correlation post-injury (in the absence of biochemical ischemia)
			In those patients without biochemical ischemia, IL-6 levels spiked in the first 48 h (*p* < 0.01)			
			IL-1b activation was less commonly observed (only 53% of measures)			

Hutchinson et al. (20)	Unclear tissue location (“frontal cortex”) IL-1a, IL-1b, IL-1ra q6 h pooled samples (mean no. samples = 9.1; range = 4–23) Isotonic central nervous system perfusate	Not specified	IL-1a and IL-1b concentrations were lower than IL-1ra A positive correlation between IL-1ra and IL-1b was seen (*p* = 0.028) No correlation between IL-1b and IL-1ra was found No correlation between cytokines and CMD glucose, glutamate, LPR	ICP: ICP was negatively correlation to IL-1ra (*p* = 0.041)	Not specified	The appears to be a correlation between IL-1ra and IL-1b There is a negative correlation between ICP and IL-1ra Mean IL-1ra levels correlate to patient outcome at 6 months
				No correlation between other cytokines and ICP		
				No correlation between cytokines and CPP		
				Outcome: significant relationship between mean IL-1ra levels and poor outcome (dichotomized GOS at 6 months) (*p* = 0.018)—high IL-1ra was associated with good outcome		
				No relationship between IL-1a and IL-1b with outcome		

Mellergard et al. (21)	Mixed locations; some patients with two catheters (unclear which patients)	Not specified	IL-1b peaked in the first 12 h period IL-6 peaked after 12 h post-insertion IL-8 peaked within the first 6 h post-insertion MIP-1b peaked within the first 6 h post-insertion FGF-2 peaked within the first 6 h post-insertion IL-10, VEGF, and RANTES did not show a temporal profile	N/A	Not specified	CMD catheter insertion leads to IL-1b/IL-6/IL-8/MIP1b within the first 6–12 h, which then decrease during the subsequent time afterward
	IL-1b, IL-6, IL-8, FGF-2, MIP-1b, RANTES, VEGF, IL-10					
	q6 h pooled samples for 36 h					
	Ringer-dextran 60 perfusate					

^c^Mellergard et al. (22)	Paired catheters (1 peri-lesonal; 1 healthy tissue)—used the catheter with highest glycerol levels for measuring cytokines IL-1b, IL-6, IL-10 q6 h pooled analysis for 7 days Ringer-dextran 60 perfusate	Not specified; various surgical procedure for hemotomas in TBI group	IL-1b increased during the first 48 h, and then decreased IL-6 increased over the first 48 h, and then decreased IL-10 remained elevated throughout the measurement period	N/A	Not specified	IL-1b and IL-6 display a peak elevation during the first 48 h post-TBI IL-10 remains elevated through the first 7 days post-TBI


^c^Mellergard et al. (23)	Paired catheters (1 peri-lesonal; 1 healthy tissue)—used the catheter with highest glycerol levels for measuring cytokines	Not specified; various surgical procedure for hemotomas in TBI group	FGF-2 levels peaked at day 3 post-TBI VEGF levels peaked on day 2 post-TBI	N/A	Not specified	FGF-2/VEGF levels peaked on days 3 and 2 post-TBI
	FGF-2, VEGF					
	q6 h pooled analysis for 7 days					
	Ringer-dextran 60 perfusate					

Mellergard et al. (24)	Unclear location IL-1b, IL-6, IL-8, FGF-2, MIP-1b, RANTES, VEGF, IL-10 q6 h pooled sample analysis Ringer-dextran 60 perfusate	Local protocols; not otherwise specified	IL-1b, IL-8, and IL-10 did not display age-related differences	N/A	Not specified	There may be an age-related difference in the expression of VEGF, MIP-1b, RANTES, and FGF-2 post-TBI
			VEGF, MIP-1b, and RANTES were different in the <25 years age group vs. over 25 years age			
			FGF-2 levels were significantly higher in the >65-year-old group (*p* < 0.0001)			

Mondello et al. (25)	Unclear location IL-1b, IL-6, TNF-a, INF-g Unclear sampling interval Unclear perfusate	Not specified	IL-6 showed high initial values that then decreased, in contrast IL-1beta, TNF-alpha and INF-gamma showed later elevations	N/A	Not specified	Variable cytokine temporal profiles are seen post-TBI
			UCH-L1 levels negatively correlated (*p* < 0.05) with IL-1beta, widely used biomarker of inflammation			

Parez-Barcena et al. (26)	Right frontal location; unclear tissue quality IL-1b, IL-6, IL-8, IL10, IL-12, TNF-a q8 h sample analysis (up to 248 h duration) Isotonic central nervous system perfusate	Varied ICP/CPP directed therapies; some use of barbiturates	IL-1b, IL-6, and IL-8 peaked during first 24 h post-injury IL-10 remained unchanged during the sampling period	ICP: no correlation between IL-1b, IL-6, IL-8 and IL-10 with ICP	Not specified	IL-1b, IL-6 and IL-8 peaked within the first 24 h post-injury No clear association was found between cytokines and ICP, PbtO_2_, CT changes
				PbtO_2_: no clear correlation between cytokines and PbtO_2_ CT: no association found between cytokines and subsequent CT defined swelling or lesion change		

Roberts et al. (27)	Healthy tissue IL-1a, IL-1b, IL-2, IL-4, IL-5, IL-6, IL-8, IL-10, and TNF-a q6 h pooled analysis (up to 156 h of monitoring) Isotonic central nervous system perfusate	Varied; one patient had DC	IL-1a, IL-1b, and TNF-a were elevated initially after injury IL-6 and IL-8 were substantially higher in the CMD compared to other cytokines IL-5 was barely detectable Similar cytokine concentrations were seen in CSF and CMD, which were both substantially higher than jugular plasma sampled Increase CMD concentrations of MMP-8 and MMP-9 were seen with increases in the levels of IL-1a, IL-2, and IL-1a and -2 and TNF-a, respectively. In contrast, the CMD levels of MMP-7 decreased with increases in IL-1b, IL-2, and IL-6	Neuro Exam: IL-1b, IL-4 and TNF-a levels were substantially higher in those with loss of pupillary reactivity	Not specified	IL-1a, IL-1b, TNF-a, IL-6 and IL-8 predominate the cytokine response post TBI Various patterns of MMP changes are seen in correlation with changes in cytokine expression IL-1b, IL-4 and TNF-a levels were higher in those with loss of pupillary reactivity IL-6 and IL-8 correlation with CPP. TNF-a correlations with ICP
				ICP: IL-2 displayed a negative correlation to ICP		
				TNF-a displayed a negative correlation to ICP		
				CPP: IL-6 and IL-8 displayed a negative correlation to CPP		
				PbtO_2_: no correlation found between cytokines and PbtO_2_		
				Outcome: n*o* correlation between cytokines and GOS		

Winter et al. (28)	Peri-lesional IL-1b, IL-6, NGF q3 h sampling (for 6 days) Normal saline perfusate	Not specified	CMD cytokine analysis is feasible and safe	Peak cytokine levels were seen within the first 36 h post-injury	None	CMD cytokine analysis is feasible IL-1b may be the predominant CMD cytokine expressed Unclear patterns in survivors vs. non-survivors
				IL-1b predominated with substantially higher concentrations compared to IL-6 and NGF		
				IL-6 was high in survivors, while NGF was lower in non-survivors		

Winter et al. (29)	Healthy tissue	Not specified	Higher IL-6 was seen in survivors (*p* = 0.04)	N/A	None	IL-6 levels in CMD samples may correlation to survival and GOS at 6 months
	IL-1b, IL-6, NGF		Peak IL-6 correlated to GOS at 6 months (*p* = 0.03)			
	q3 h sampling		Peak NGF:IL-1b ratios were significantly lower in survivors (*p* = 0.01)			
	Normal saline perfusate					

*TBI, traumatic brain injury; sTBI, severe TBI; GOS, Glasgow outcome scale; CMD, cerebral microdialysis; RCT, randomized control trial; ICP, intracranial pressure; CT, computed tomography; PbtO_2_, brain tissue oxygen monitoring; CPP, cerebral perfusion pressure; CSF, cerebrospinal fluid; LPR, lactate:pyruvate ratio; DC, decompressive craniectomy; μmol, micromolar; mm Hg, millimeters of mercury; L, liter; μmol, micromolar; IL, interleukin; a, alpha; b, beta; g, gamma; TNF, tumor necrosis factor; INF, interferon; MCP, monocyte chemoattractant protein; MIPs, macrophage inflammatory proteins; TGF, transforming growth factor; NGF, nerve growth factor; BDNF, brain-derived neurotrophic factor; GDNF, glial-derived neurotrophic factor; TNFR, tumor necrosis factor receptor; GM-CSF, granulocyte macrophage colony stimulating factor; sVCAM, soluble vascular cell adhesion molecule; sICAM, soluble intracellular adhesion molecule; PDGF, platelet-derived growth factor; VEGF, vascular endothelial growth factor; MDC, macrophage-derived chemokine; FGFR, fibroblast growth factor receptor*.

*^a^Same patient population reported in both Helmy et al. (14) and Helmy et al. (15)*.

*^b^Same patient population described in Helmy et al. (16) and Helmy et al. (17)*.

*^c^Same patient population reported in both Mellergard et al. (22) and Mellergard et al. (23)*.

**Table 4:** CSF cytokine measures and outcomes.

Reference	Interval of cytokine measure	Measured CMD cytokines	Interventional therapies applied during measurement	Outcome(s) of interest (patient outcome, neurophysiologic outcome, tissue outcome)	Other outcomes	Conclusions
Abboud et al. (30)	q12-h Intervals for 5 days	IL-1α,IL-1β, IL-2, IL-4, IL-5, IL-6, IL-8, IL-10, IL-13, MIP-1a, MIP-1b, TNF-a, VEGF	Not specified	GOS at 6 and 12 months post-injury Statistically significant differences in IL-4, IL-5, IL-6, IL-8, IL-13, and TNF-α (all *p* < 0.05) were observed between TBI survivors vs. non-survivors over 5 days	N/A	Elevated IL-4, IL-6, IL-8, IL-23, and TNF-a levels may be associated with poor outcome at 6 and 12 months
						Similarly, low IL-5 and IL-13 may be associated with poor outcome

Bell et al. (31)	q24-h Intervals for 3 days	IL-6, IL-10	High variable; barbiturates and various ICP/CPP-directed therapies	Mortality (at unclear interval)	IL-6 and IL-10 levels were increased compared to controls	Elevated IL-10 levels may be associated with mortality
				IL-6 is not associated with mortality		
	Control group had banked CSF			IL-10 is associated with mortality (*p* = 0.022)		

Chiaretti et al. (32)	At 2 and 48 h post-injury Controls were initially investigated for meningitis but were found to be negative. CSF was banked	IL-6, NGF	Highly protocolized therapy; seemingly homogenous between patients	GOS at 6 months	IL-6 and NGF were both elevated and increased between the two sampling periods	Lower IL-6 and NGF levels early post-TBI may be associated with better outcome at 6 months
				Low IL-6 and NGF at 2 h post-injury was associated with good outcome (*p* < 0.01) Increased IL-6 variation between the two time points was correlated with better outcome		
					IL-6 and NGF were positively correlated at both time periods	

Chiaretti et al. (33)	At 2 and 48 h post-injury	IL-1b, IL-6, NGF, BDNF, GDNF	Highly protocolized therapy; seemingly homogenous between patients	GOS at 6 months Low NGF at 2 h (*p* < 0.01) and high NGF/IL-6 (*p* = 0.02/*p* < 0.01) at 48 h were associated with better outcome	N/A	Low initial NGF, followed by increased NGF/IL-6 may be associated with good outcome at 6 months
						Low IL-1b at 48 h may be associated with better outcome at 6 months
				Low IL-1b at 48 h was associated with better outcome (*p* < 0.01)		

Chiaretti et al. (34)	At 2 and 24 h post-injury	IL-1b and IL-6	Highly protocolized therapy; seemingly homogenous between patients	Dichotomized GOS at 6 months (good = 4 or 5; poor = 3 or less)	IL-1b and IL-6 at 2 h were higher in the TBI cohort	Elevated IL-1b and IL-6 at both 2 and 24 h post-injury may be associate with poor outcome at 6 months
				Higher CSF IL-1b and IL-6 at both 2 h and 24 h were seen in those patients with poor outcome at 6 months		

Hans et al. (35)	Daily CSF samples up to 21 days post-injury	IL-6 and sIL-6R	Not specified	Dichotomized GOS at 6 months (good = 4 or 5; poor = 3 or less)	CSF levels of IL-6 and sIL-6R were higher than compared to plasma	Elevated IL-6/sIL-6R may be associated with poor outcome at 6 months
				High IL6/sIL-6R was associated with poor outcome at 6 months		

Hayakata et al. (36)	6, 12, 24, 48, 72, and 96 h after injury	TNF-a, IL-1, IL-6, IL-8, and IL-10	Varied therapies; hypothermia and other ICP/CPP-directed approaches	Dichotomized GOS at 6 months (good = 4 or 5; poor = 3 or less) CSF IL-1b was found to be higher in those with poor outcome	ICP: IL-1b was significantly positively correlated with ICP throughout the entire study (*p* < 0.05) Various cytokine elevations were seen during S100B elevations. IL-1b peaks was correlated with S100B peak (*p* < 0.005)	Elevated IL-1b may be associated with poor outcome at 6 months Elevated IL-1b may be associated with elevated ICP

Jamil et al. (37)	Unclear interval; “acute” period post-TBI	IL-1b, IL-4, IL-5, IL-6, IL-7, IL-8, IL-10, IL-12, TNF-a, sICAM-1, sVCAM-1, sFAS	Not specified	Patient health questionnaire (PHQ-9) at 6 and 12 months post-injury	N/A	Elevated IL-6 and IL-8 may be associate with depression at 6 TNF-a, IL-4, and IL-1b may be associated with lower chance of depression at 12 months
				Acute CSF IL-6 (*p* = 0.008), IL-8 (*p* = 0.034), and ICAM1 (*p* = 0.025) levels were higher among patients who would go on to develop depression 6 months after injury		
				Acute CSF TNF-a (*p* = 0.036), IL-4 (*p* = 0.007), and IL-1b (*p* = 0.001) levels were individually associated with lower depression risk at 12 months post-injury		

Juengst et al. (38)	Within first week of injury	IL-4, IL-5, IL-8, IL-12, TNF-a, sVCAM, sICAM	Not specified	Apathy subscale of the frontal systems behavior scale, collected at 6 and 12 months post-TBI	N/A	Higher acute CSF IL5, sVCAM, and sICAM with apathy at 6 months and lower acute serum TNFalpha, IL8, and IL5 with apathy at 12 months
				Higher acute CSF IL5, sVCAM, and sICAM with apathy at 6 months and lower acute serum TNFalpha, IL8, and IL5 with apathy at 12 months (*p* < 0.05)		

Juengst et al. (39)	2 times daily for 6 days	TNF-a	Not specified	At 6 and 12 months post-injury, FrSBe disinhibition subscale; suicidal endorsement was assessed by the PHQ-9	TBI patients had significantly higher CSF TNF-a levels compared to controls	Acute levels of TNF-a may correlate to 6 and 12 month rates of disinhibition
				No relationship between TNF-a in CSF and suicidality at 6 or 12 months		
				Acute serum TNFa levels were inversely associated with 12-month disinhibition (*r* = 0.520, *p* = 0.027) and achieved borderline significance with 6-month disinhibition (*r* = 0.470, *p* = 0.057)		

Juengst et al. (40)	2 times daily up to 6 days	IL-1β, IL-4, IL-5, IL-6, IL-7, IL-8, IL-10, IL-12, TNF-a, sVCAM-1, sICAM-1, sFAS	Not specified	PHQ-9 was administered to participants at 6 and 12 months after injury	IL-1β, IL-4, IL-6, IL-7, IL-8, IL-10, TNF-α, sVCAM-1, sICAM-1, and sFAS (*p* < 0.05) were significantly elevated compared to controls	Elevated sVCAM-1, sICAM-1 and sFAS may be associated with PTD at 6 months Elevated IL-7 and IL-8 may be associated with PTD at 12 months
				The inflammatory cell surface markers sVCAM-1, sICAM-1, and sFAS in the CSF were each positively associated with PTD at 6 months (*p* < 0.02 for all comparisons).		
				The cytokine IL-8 was positively associated with PTD at 12 months (*p* < 0.02), while the cytokine IL-7 was inversely associated with PTD at 12 months (*p* < 0.05)		

Kirchhoff et al. (41)	Upon EVD insertion, then at 12, 24, and 48 h post-injury	IL-10	Not specified	Mortality at unspecified interval IL-10 was significantly higher in non-survivors	IL-10 was higher at all time points compared to non-TBI controls	Elevated CSF IL-10 at admission was associated with mortality
	Control group: CSF gained from spinal anesthetics in elective non-TBI surgical cases				

Kossmann et al. (42)	q24 h for unclear duration Control group: non-TBI patients (1 VPS and 2 Dx LP)	IL1b, IL-6, TNF-a, NGF	Various therapies; heterogeneous across population	Dichotomized GOS at 3 months (good = 4 or 5; poor = 3 or less)	IL-6 and NGF were high in TBI patients compared to control samples	NGF may be elevated in those with good outcome
				High IL-6 levels were associated with NGF presence in CSF		
				NGF levels were elevated in those with better outcomes		

Kumar et al. (43)	2 times daily for 5 days	IL-6	Not specified	Dichotomized GOS at 6 and 12 months (good = 4 or 5; poor = 3 or less)	IL-6 levels were higher in TBI compared to controls	High IL-6 during the first 5 days of injury may be associated with poor outcome at 6 months
				Association between high IL-6 upon admission and 6-month GOS (*p* = 0.003)		

Kumar et al. (44)	2 times daily for up to 5 days	IL-1β, IL-4, IL-5, IL-6, IL-7, IL-8, IL-10, IL-12, TNF-a, sVCAM-1, sICAM-1, sFAS	Not specified	Trichotomized GOS at 6 and 12 months (good = 4 or 5; poor = 3 or 2; dead = 1)	Cytokines were elevated in TBI patients compared to controls	Elevated IL-5, IL-6, IL-8, IL-10, sVCAM-1, and sICMA-1 may be associated with poor outcome at 6 months
				Individuals in cluster 1 (increased sICAM-1, sFAS, IL-10, IL-6, sVCAM-1, IL-5, and IL-8) had a 10.9 times increased likelihood of GOS scores of 2/3 vs. 4/5 at 6 months compared to cluster 2 (increased IL-12, IL7, IL-4)		

Kushi et al. (45)	Admission, 24, 72, and 168 h post-injury	IL-6, IL-8	High protocolized treatment; fairly homogeneous therapy	Mortality at unspecified interval	N/A	Elevated IL-6 and IL-8 during the first week post-TBI may be associated with mortality
				IL-6 and IL-8 levels were significantly higher in CSF compared to serum		
				IL-6 and IL-8 levels were significantly higher in non-survivors		

Nwachuku et al. (46)	q6 h for 5 days	IL-1b, IL-6, TNF-a, IFN-a, IL-12p70, IL-10, and IL-8	Not specified	Dichotomized GOS at 3, 6, 12, and 24 months (good = 4 or 5; poor = 3 or less)	N/A	Elevated mean 5-day levels of various cytokines may be associated with poor outcome at 3, 6, 12, and 24 months post-injury
				Mean 5-day levels of IFN-a, IL-10, IL-12 p70, IL-1, IL-6, IL-8, and TNF-a were associated with outcome (*p* < 0.05)		

Santarseiri et al. (47)	2 times daily for up to 6 days	IL-1β, IL-4, IL-5, IL-6, IL-7, IL-8, IL-10, IL-12, TNF-a, sVCAM-1, sICAM-1, sFAS	Not specified	Dichotomized GOS at 6 months (good = 4 or 5; poor = 3 or less)	N/A	Low mean IL-6, IL-8, IL-10, sICAM-1, and TNF-a may be associated with good outcome at 6 months post-injury
				Cortisol: high cortisol patients were more likely to have elevated IL-10, IL-1b, IL-6, sFas, sICAM-1, sVCAM-1 and TNFa (*p* < 0.01 all comparisons, except IL-1b, *p* < 0.05) compared to low cortisol patients		
				Outcome: significant associations between GOS and mean levels of IL-10, IL-6, IL-8, sFas, sICAM-1 (*p* < 0.01) and TNF-a (*p* < 0.05), with lower levels associated with favorable outcome		

Shiozaki et al. (48)	q6 h for unclear duration	IL-1b, IL-1ra, IL10, TNF-a, sTNFr-I	Highly Protocolized therapy	Dichotomized GOS at 6 months (good = 4 or 5; poor = 3 or less)	IL-1b, IL-1ra, sTNFr-I, and IL-10 were significantly higher in patients with high ICP than those with low ICP (*p* = 0.002, *p* = 0.006, *p* = 0.009, and *p* = 0.009, respectively). However, the CSF concentrations of TNF-a did not differ between patients with high ICP and those with low ICP	Elevated IL-lb, IL-1ra, IL-10, and sTNFr-I may be associated with poor outcome at 6 months Elevated IL-1b, IL-1ra, IL-10, sTNFr-I may be associated with high ICP
				IL-1b, IL-1ra, sTNFr-I, and IL-10 were significantly higher in patients with an unfavorable outcome than in patients with a favorable outcome (*p* = 0.006, *p* = 0.009, *p* = 0.003, and *p* = 0.012, respectively)		
Singhal et al. (49)	Unclear interval	IL-1b, IL-6	Not specified	*SSEP: positive* correlation between IL-6 and SSEP96 (mean change in SSEP over 96 h) (*p* = 0.0133)	N/A	Elevated IL-6 may be positively correlated to SSEP over the first 96 h Peak IL-6 levels may be associated with outcome at 3 months
				Outcome: GOS at 3 months		
				Peak IL-6 levels were associated with good outcome (*p* = 0.026)		

Whalen et al. (50)	Unclear sampling intervals	IL-8	Not specified	Mortality at unspecified interval	IL-8 levels were elevated compared to controls	Elevated IL-8 levels during the first week of injury may be associated with mortality
				Elevated CSF IL-8 levels were associated with mortality (*p* = 0.01)		
**Neurophysiologic association**						

Muller et al. (51)	Daily for 7 days	IL-6, IL-8, IL-10	Not specified	Transcranial doppler (TCD)-defined cerebral blood flow velocity	N/A	Elevated IL-6 and IL-8 in the first 7 days may be negatively correlated to TCD defined MCBFV
				Mean IL-6 and IL-8 level were significantly correlated to MCBFV (*r* = −0.341 and −0.361, respectively; *p* < 0.05)		

Stein et al. (52)	2 times daily for 7 days	IL-1b, IL-6, IL-8, IL-10, and TNF-a	High protocolized therapy	ICP: negative association between early (within first 12 h of injury) IL-6 and ICP (*p* = 0.004)	N/A	Elevated IL-6 within the first 12 h of injury may be associated with low ICP Elevated IL-8 levels may be associated with low CPP
				Positive correlation between time spent with CPP below 60 mm Hg and IL-8 levels (*p* = 0.001)		
				Outcome: dichotomized GOSE at 6 months (good = 5–8; poor = 1–4)		
				No association between CSF cytokines and outcome		
**Nil association studies**						

Amick et al. (53)	Unclear time frame post-TBI (from 4 to 122 h after injury)	IL-2, IL-4, IL-6, IL-12	Highly variable; barbiturates and various ICP/CPP-directed therapies	GOS at 6 months post-Injury	IL-6 and IL-12 were increased compared to control group	No association between IL-2, IL-4, IL-6, and IL-12 with GOS at 6 months
	Banked samples from a non-TBI control group (*n* = 12); CSF gained from investigations for meningitis			No correlation between measured CSF cytokines and GOS		

Buttram et al. (54)	Collected 18, 24, 48, and 72 h post-injury	IL-1a, IL-1b, IL-2, IL-4, IL-5, IL-6, IL-7, IL-8, IL-10, IL-12p70, IL-13, IL-15, IL-17, IP-10, eotaxin, TNF-a, INF-g, MCP-1, MIP-1a	Not well specified; half the groups was subjected to moderate hypothermia for 48 h (32–33°C)	Dichotomized GOS at 6 months	Cytokine levels in TBI patients were significantly higher compared to controls	There is no association between CSF cytokines and outcome at 6 months
				No association between CSF cytokines and outcome		

Csuka et al. (55)	Daily until EVD removal	IL-6, IL-10, TNF-a, TGF-B1	Unclear ICP/CPP-directed therapies	Outcome: GOS at 3–6 months No correlation found between cytokines and outcome ICP: no correlation between cytokines and ICP	IL-10 was found in both CSF and serum during the measurement period	CSF cytokines do not correlate to outcome at 3–6 months
						CSF cytokines do not correlate to ICP

Diamond et al. (56)	q12 h for 6 days	IL1b	Not specified	EEG and Epileptologist defined PTE	Serum IL-1b levels was associated with PTE	CSF IL-1b levels within the first week of injury is not associated with PTE
				CSF IL-1b was not statistically associated with PTE		

Goodman et al. (57)	Unclear sampling interval	IL-1, IL-6, IL-8, IL-10, IL-12, TNF	Not specified	ICP/CPP: no correlation between CSF cytokines and ICP or CPP	Serum and CSF IL-6 and IL-8 were both elevated consistently	CSF cytokines are not associated with changes in ICP and CPP

Gopcevic et al. (58)	Unclear sampling interval	IL-8	Not specified	30-day in-hospital mortality: no correlation between CSF IL-8 levels and patient outcome	No correlation between plasma and CSF IL-8 levels	CSF IL-8 is not associated with in-hospital mortality at 1 month

Lenzlinger et al. (59)	Daily for unclear duration	sIL-2R, B2M, neopterin	Unclear ICP direct therapy	GOS at 4–6 months	Neopterin levels were higher in CSF than serum	sIL-2R, B2M, and neopterin in CSF have no correlation to outcome at 4–6 months
				No association between measured cytokines and outcome	B2M and sIL-2R levels were higher in serum	

Maier et al. (60)	Admission and daily up to day 10	sTNFRp55, sTNFRp75	Not specified	GOS at 6 months	sTNRFp55 and STNFRp75 is elevated in CSF compared to control	sTNFRp55 and sTNFRp75 CSF levels are not associated with outcome at 6 months
				No correlation between sTNFRp55 or sTNFRp75 and outcome		

Maier et al. (61)	Admission and daily up to day 14	IL-6, IL-8, IL-10	Not specified	Mortality at unspecified interval No correlation between CSF cytokines and patient outcome	IL-6 and IL-8 were directly correlated with each other with CSF level higher than serum	CSF IL-6, IL-8, and IL-10 levels do not correlated with mortality
					All measured cytokines were higher in TBI patients compared to controls	

Morganti-Kossmann et al. (62)	Unclear sampling interval	IL-2, IL-4, IL-6, IL-10, TNF, IFN, GM-CSF	Not specified	Unclear outcome scale at unspecified interval	IL-6 is higher in focal injury patterns	CSF cytokines are not associated with patient outcome
				No clear association between CSF cytokines and outcome		

Newell et al. (63)	q12–24 h for 7 days	sIL-2Ra	Not specified	Dichotomized GOS at 6 months (good = 4 or 5; poor = 3 or less)	sIL-2ra levels during the measurement period were no different between TBI and controls	sIL-2Ra isn’t significantly elevated post-TBI and does not correlate with outcome
				No association between sIL-2Ra and outcome		

Ross et al. (64)	Unclear sampling interval	TNF-a	Not specified	GOS at 6 months No correlation between TNF-a and outcome	TNF-a in CSF and serum were both elevated	CSF TNF-a displayed no association with patient outcome at 6 months

Uzan et al. (65)	At 6–10, 20–28, 40–56, and 64–74 h post-injury	IL-8	Unclear ICP/CPP-directed therapies	GOS at unspecified interval	N/A	CSF IL-8 level within the first 2–3 days are not associated with outcome
				No correlation between CSF IL-8 and patient outcome		

*TBI, traumatic brain injury; sTBI, severe TBI; GOS, Glasgow outcome scale; CMD, cerebral microdialysis; RCT, randomized control trial; ICP, intracranial pressure; CT, computed tomography; PbtO_2_, brain tissue oxygen monitoring; CPP, cerebral perfusion pressure; CSF, cerebrospinal fluid; LPR, lactate:pyruvate ratio; DC, decompressive craniectomy; μmol, micromolar; mm Hg, millimeters of mercury; L, liter; μmol, micromolar; IL, interleukin; a, alpha; b, beta; g, gamma; TNF, tumor necrosis factor; INF, interferon; MCP, monocyte chemoattractant protein; MIPs, macrophage inflammatory proteins; TGF, transforming growth factor; NGF, nerve growth factor; BDNF, brain-derived neurotrophic factor; GDNF, glial derived neurotrophic factor; TNFR, tumor necrosis factor receptor; GM-CSF, granulocyte macrophage colony stimulating factor; sVCAM, soluble vascular cell adhesion molecule; sICAM, soluble intracellular adhesion molecule; PDGF, platelet-derived growth factor; VEGF, vascular endothelial growth factor; MDC, macrophage derived chemokine; FGFR, fibroblast growth factor receptor*.

### Bias Assessment

As the goal of this review was to produce a systematically conducted scoping review of the available literature on CMD and CSF cytokine measures in sTBI, formal bias assessment was not done. Our desire was to produce a comprehensive overview of the current literature on the topic of CMD/CSF cytokines in sTBI. Formal evidence grading was not conducted (given the limited and heterogenous literature body), and thus we deemed formal bias risk assessment unnecessary for this emerging area of literature, which clearly suffers from standard biases associated with new areas of clinical research.

### Statistical Analysis

A meta-analysis was not performed in this study due to the heterogeneity of data and study design within the articles identified.

## Results

### Search Strategy Results

#### CMD Cytokine Search

**Figure 1:**
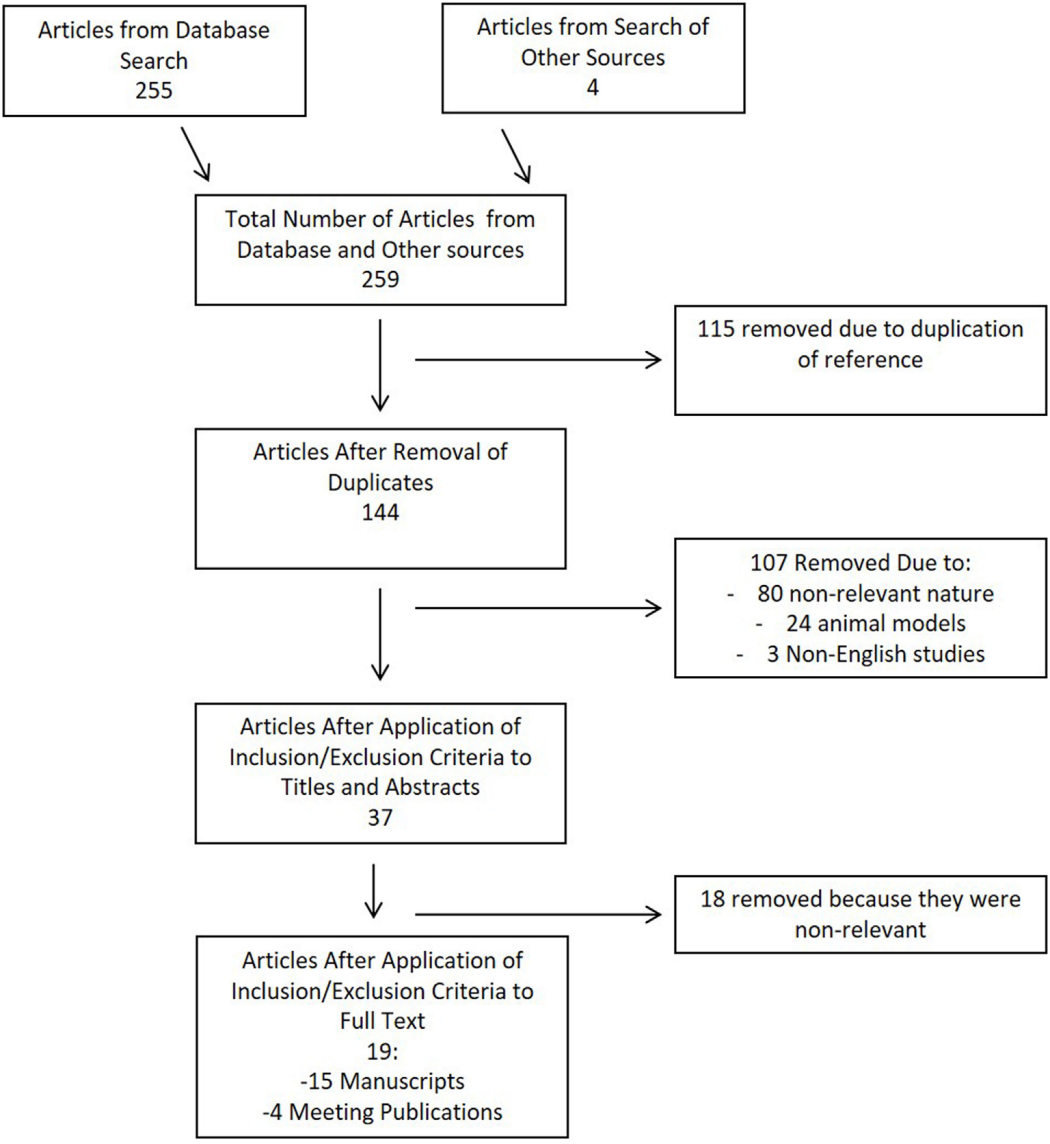
Flow diagram of search results for cerebral microdialysis review.

Results of the search strategy for CMD cytokines in sTBI is shown in the flow diagram in Figure 1. In total, 259 articles were identified, with 255 from the database and 4 from meeting proceeding sources. After removal of the duplicates, there were 144 articles left for assessment in the first filter of title and abstract. Thirty-seven articles passed the first filter, requiring acquisition of the full manuscript to assess inclusion eligibility. After assessing the full manuscripts, 19 articles were deemed eligible for final inclusion in the scoping systematic review. No articles were added from the reference sections of either review papers or the parent manuscripts included in the systematic review.

#### CSF Cytokine Search

**Figure 2:**
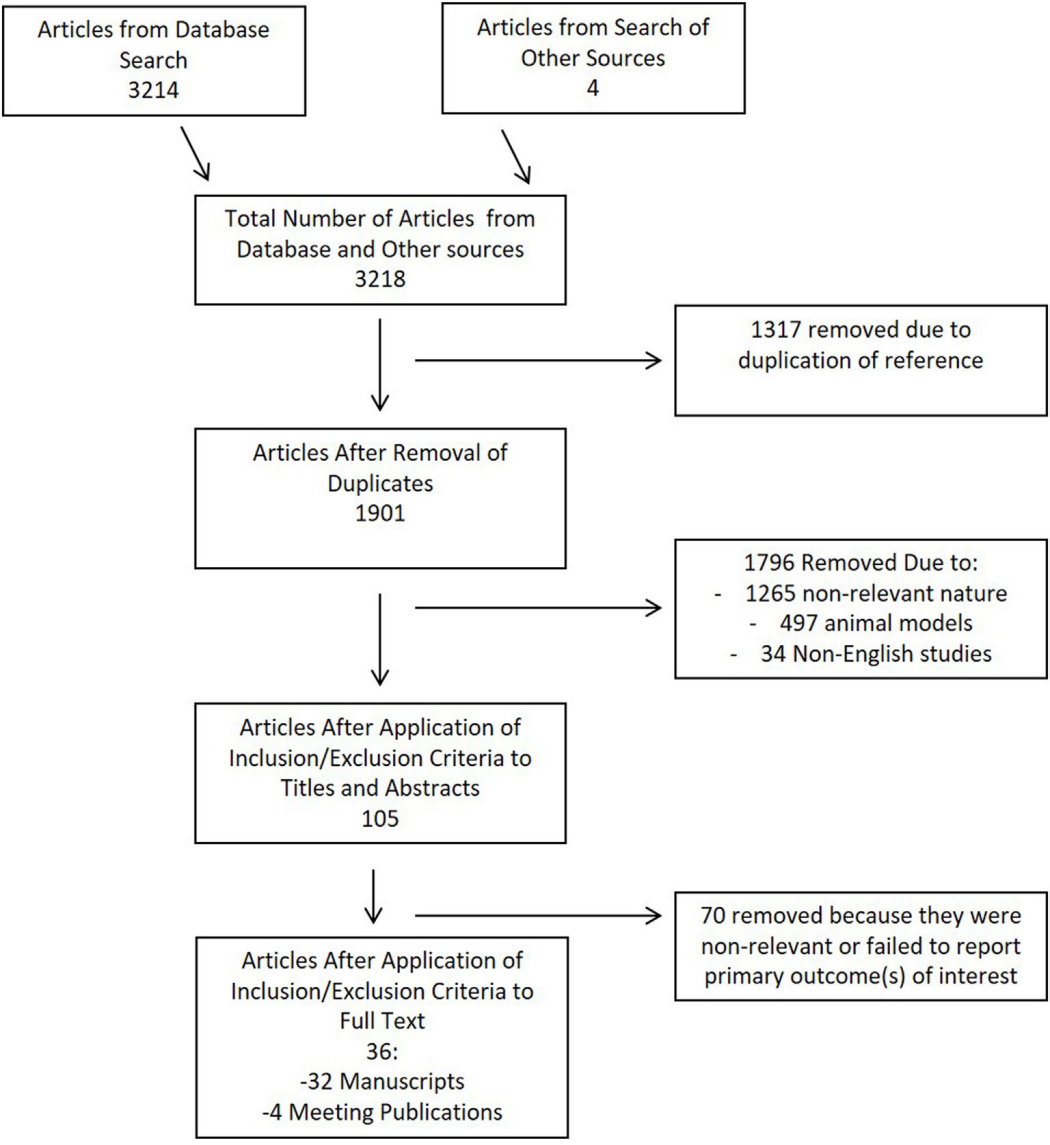
Flow diagram of search results for cerebrospinal fluid review.

The search strategy flow diagram for the CSF cytokine scoping systematic review is shown in Figure 2. Overall, 3,218 articles were identified, with 3,214 from the database search and 4 from published meeting proceedings. There were 1,317 duplicates removed, leaving 1,901 references to review in the first filter. Applying the inclusion/exclusion criteria to the title and abstract of these articles, 105 manuscripts were selected for review of the full article. One additional reference was added from the reference sections of review papers. During the second filter of the full manuscript, 36 met the final inclusion criteria for the scoping systematic review. Remaining articles were excluded due to non-relevance.

### Patient/Study Demographics

#### CDM Cytokine Review

Of the 19 articles included in the CMD cytokine portion of the systematic review (11–19), 15 were formal manuscript publications (14–24, 26–29) and 4 were meeting abstract publications (11–13, 25). There were 13 prospective studies (13–16, 18–21, 25–29), with 12 prospective observational studies (13–15, 18–21, 25–29) and 1 prospective randomized control trial (16). Four studies were retrospective case series or database reviews (11, 20, 22, 23). Finally, two studies were of “unknown” study design given a lack of information available within the methods (12, 24).

The study population described in CMD cytokine papers was generally poorly characterized sTBI patient populations, undergoing various ICU and surgical therapies for their heterogeneous intracranial pathology (11–15, 18–25, 27–29). Three studies focused on only those patients with imaging defined “diffuse” brain injury, without extra-axial or large focal intraparenchymal lesions (16, 17, 26).

A total of 267 unique patients with sTBI were described across the 19 studies included in the CMD cytokine review. Thirty-six patients were “diffuse” sTBI only (16, 17, 26), with the remaining being unspecified heterogeneous sTBI pathology. We believe that some of the studies included within this portion of the review may contain duplicate patient information, as marked in Tables 1 and 3. Multiple publications from the same research groups likely were conducted on the same patient populations, yielding unique and separate manuscripts on the same group of patients. Though we must acknowledge it was difficult to determine, in some circumstances, whether CMD cytokine analysis was being conducted on new patient groups or existing banked samples from previous prospective studies. With that said, our goal for the CMD cytokine scoping review was to provide an overview of all available literature in the area, hence we have included all published papers on CMD cytokines in sTBI within this review.

#### CSF Cytokine Review

Of the 36 articles included in the CSF cytokine systematic review (20–65), 32 were formal manuscript publications (30–36, 39, 40, 42–61, 63–65) and 4 were meeting abstract publications (37, 38, 41, 62). There were 34 prospective studies, all being observational studies (30–61, 64, 65). One study was a retrospective case series (63). Finally, one study had insufficient information to determine the design (62).

The populations described with in the CSF cytokine studies were almost all sTBI patients with unspecified heterogeneous injury patterns. Three studies documented the inclusion of both moderate-severe patients within the methods (39, 53, 62). We were unable to separate the moderate and sTBI patients within these studies, hence they were all included in the final descriptive statistics.

A total of 1,363 patients were described across all studies included in the CSF cytokine systematic review. The mean age for each study cohort varied significantly across studies. Twenty-one studies included pediatric patients within their studies, either as the primary population of interest or included with adult patients (31–35, 42, 44, 47–50, 52, 54, 55, 57, 59, 60, 63–65). Therapies received by these patients while in the ICU varied significantly, with profound heterogeneity in treatment provided. Details surrounding patient cohort, study design, and concurrent therapies can be found in Tables 2 and 4. We made substantial efforts to exclude duplicate patient data across studies. However, given that many of the papers came from centers of excellence for TBI research, some of the patient data may be cross reported in multiple studies. This could reduce the total overall number of unique patients. It was impossible based on the information provided within the parent studies to tease out all patients which were reported more than once.

### Cytokine Measurement Technique

#### CMD Cytokine Review

Location of the CMD catheter was the following: mixed healthy/peri-lesional tissue in six studies (11, 13, 15, 21–23), peri-lesional in six studies (14, 16–19, 28), healthy tissue in two studies (27, 29), and unknown tissue location in five studies (12, 20, 24–26). Some studies utilized paired microdialysis catheters, one in healthy and one in peri-lesional tissue (13, 15, 22, 23). One study evaluated two catheters in one location (18). Analysis interval for CMD samples was as follows: every 6 h in 12 studies (14–24, 27), every 8 h in 1 study (26), every 3 h in 2 studies (28, 29), and unspecified in 4 studies (11–13, 25). The duration of sample collection varied as well, with the typical collection period of 5–7 days.

Numerous different panels of cytokines were evaluated within the CMD samples, across the studies included within the review. The most commonly studied cytokines included IL-1b, IL-1ra, IL-6, IL-8, and IL-10. Details of CMD technique and catheter locations are listed in Table 3.

#### CSF Cytokine Review

Sampling of CSF was conducted through external ventricular drains (EVDs) in almost all patients described within the studies included in the CSF cytokine systematic review (30–65). Sampling and analysis frequency varied significantly from study to study with sampling occurring from every 6 h to daily. Duration of sampling varied as well, up to 21 days post-injury (35).

Like the CMD cytokine papers, the CSF cytokine papers included in this review reported the measurement of various cytokines. The most commonly measured cytokines in CSF reported were IL-1b, IL-1ra, IL-6, IL-8, IL-10, and TNF. The details of CSF sampling and specific cytokines measured can be found in Table 4.

### Outcomes

#### CMD Cytokine Review

Given that the CMD cytokine portion of this review was a scoping review of all the literature on CMD cytokines in sTBI, the goals and outcomes reported by the studies were heterogenous, and are listed in Table 3.

Only one study described an intervention during the assessment of CMD cytokines. This study was a prospective RCT describing the application of subcutaneous rhIL-1ra post severe diffuse TBI (16). The results described both elevated CMD IL-1ra levels and a reduction in MDC in the IL-1ra treated group. The follow-up retrospective statistical analysis of all CMD measured cytokines described a trend toward an increase in M1-microglia related cytokine activation following administration of rhIL-1ra (17).

Three studies reported the correlation between CMD cytokines and patient outcome (11, 20, 29). Two studies reported a positive association between elevated CMD IL-6 and improved survival, with one describing improved Glasgow Outcome Scale (GOS) at 6 months (*p* = 0.03). One study reported the negative correlation between CMD IL-1ra and poor GOS at 6 months (*p* = 0.018).

Most studies reported the CMD cytokine profile post-TBI and temporal fluctuations (12, 14, 15, 21, 24, 26, 27). Given the myriad of cytokines measured across the studies, it is impossible to describe all of the relationships. Highlighted details can be found in Table 3. The main findings included elevated IL-1b, IL-6, and IL-8 within the first 48–72 h post-injury, with these cytokines also displaying peaks during these times (21–23). The CMD IL-10 levels were found to be more uniformly elevated during the sampling periods (22, 26). Finally, some coexpression relationships were found between IL-1b with TNF, IL-1ra with IL-1a, and MIP-1a with MIP-1b (14).

Two studies evaluated the CMD cytokine profile associated with secondary events while in the ICU (18, 19). CMD IL-6 levels were positively associated with episodes of ischemia/metabolic stress, as defined by a lactate:pyruvate ratio greater than 30 and glutamate levels greater than 80 μmol/L.

The relationship of catheter location to CMD cytokine levels was discussed in a couple of papers, with peri-lesional tissue displaying higher cytokine expressions than distant or healthy tissue locations (11, 13). Evaluation of catheter technology (18) and cytokine measure feasibility (28) were also described in a few studies.

#### CSF Cytokine Review

The 36 papers included in the CSF systematic review (30–65) included both manuscripts, which reported positive associations between CSF cytokine levels and neurophysiologic or patient outcome (30–52), and studies reporting no association (53–65) (i.e., “nil association”) between CSF cytokines and the outcomes of interest for the CSF cytokine systematic review. No studies reported an association, “nil” or otherwise, between CSF cytokine measures and tissue outcome as assessed by follow-up neuroimaging. The subsections below describe more details of these outcomes of interest, with further information found in Table 4.

##### Positive Association Studies

Twenty-three papers included within the CSF cytokine review found associations between cytokine levels and both neurophysiologic and patient outcomes. Twenty-one described the association between CSF cytokines and patient outcome (30–50). Five papers discussed the association between CSF cytokine measures and neurophysiologic outcomes (36, 48, 49, 51, 52).

###### Patient Outcome

Cerebrospinal fluid levels of several cytokines were related to functional patient outcomes. The most common outcomes specified were: overall mortality or GOS at 6–12 months post-injury. The strongest relationships between cytokines and patient outcome were for IL-1b, IL-1ra, IL-6, IL-8, IL-10, and TNF.

A strong positive correlation between CSF measured IL-6 and IL-8 with poor GOS was the most commonly described relation between CSF cytokines and patient outcome (30, 32, 35, 36, 42–47, 49, 50). Similarly, a strong association between elevated CSF measured IL-10 and poor patient outcome was described in five studies (31, 41, 46–48). Elevated CSF IL-1b was found to be associated with mortality and worse GOS at 6 months in four studies (33, 34, 36, 49). Finally, CSF TNF-alpha (TNF-a) levels were found to be associated with worse patient outcome in two studies (30, 46).

The relationship between CSF cytokine levels and neuropsychiatric outcome was described in four studies (37–40). These associations included: higher IL-6 and IL-8 were associated with a higher incidence of depression at 6 months (37), TNF-a levels with depression at 12 months (37), IL-5/IL-8/IL-12/TBF with apathy at 12 months (38), TNF-a with disinhibition at 12 months (39), and sVCAM/sICAM/sFAS with depression at 6 months (40).

###### Neurophysiologic Outcome

Three studies discussed the correlation between CSF cytokine levels and ICP/CPP (36, 48, 52). Elevated IL-6 and IL-8 levels were associated with increased ICP and decreased CPP in one study (52). Elevated CSF IL-1b was associated with increased ICP in two studies (36, 48). One study found an association between CSF IL-6 and IL-8 levels and reduced middle cerebral artery (MCA) CBFV (51). Finally, one study found an association between IL-6 levels and the mean change in somatosensory evoked potential over 96 h recording window (49).

##### Nil Association Studies

Our review identified 13 studies documenting a “nil association” between CSF measured cytokines in sTBI patients and various outcomes of interest (53–65). Eleven studies reported no association between various CSF cytokines and patient outcome, as reported by in-hospital mortality or GOS at 3–6 months (53–55, 58–65). The cytokines reported within these studies varied significantly, with the most common “nil associations” reported for IL-1b, IL-6, IL-8, IL-10, TNF-a, and sTNFR. A total of 376 patients were described within these studies. Two studies reported no association between CSF cytokine measures and ICP/CPP (55, 57), while one study failed to determine an association between CSF IL-1b and post-traumatic epilepsy (56). Further detail on the “nil association” studies can be found at the bottom of Table 4.

### Complications

Within the CMD cytokine manuscripts, the majority failed to report whether complications were considered within the data collection. Only three papers disclosed complication reporting (18, 28, 29), with two reporting “no complications” (28, 29), and one reporting a CMD catheter malfunction in one patient (19). The complication profiles may be under-reported within the CMD studies. Complication reporting within the CSF cytokine studies was essentially non-existent, with the focus of these studies the association between CSF cytokine measures and various outcomes.

## Discussion

### CMD Cytokines in sTBI

Our scoping systematic review completed for CMD cytokine measures in sTBI allows limited conclusions. Despite 19 publications (11–29), this literature is based on very small numbers of patients with many studies conducted on the same patient populations with banked CMD samples. However, the limited conclusions are important. First, CMD-based measurement of cytokines is feasible. Second, CMD catheter location makes a difference in the levels of cytokines measured, with peri-lesional tissue producing high levels compared to distant or healthier tissue (11, 13). Third, peaks in CMD cytokine measures may occur within the first 48–72 h for IL-1b, IL-6, and IL-8 (21–23). Interestingly, IL-10 seems to remain elevated in CMD samples through the duration of the sampling periods described (22, 26). Fourth, IL-6 levels may prove to be predictive of ongoing second insults such as ischemia (18, 19). Fifth, the data from the rhIL-1ra studies (16, 17) shows that subcutaneous rhIL-1ra leads to both an increase in CMD IL-1ra and a modulation of microglial/macrophage based cytokine profiles. Sixth, CMD IL-1b/IL-1ra/IL-6/IL-8 may be associated with poor outcome (11, 20, 29), up to 6 months post-injury. Finally, complications related to the use of CMD catheters are likely to be under-reported.

### CSF Cytokines in sTBI

Our systematic review of CSF cytokines in sTBI, focused on the association between cytokine measures and patient, tissue outcome, or neurophysiology outcomes identified some interesting trends. First, a large number of heterogeneous studies correlated CSF cytokine levels with patient outcome, defined as either mortality or GOS at 6–12 months post-injury. Various large panels of cytokines were described within these studies, but the strongest associations with outcome were found for IL-1b, IL-1ra, IL-6, IL-8, IL-10, and TNF. Most studies described an association between elevated levels of these cytokines and poor GOS/increased mortality. Second, psychiatric outcome at 6–12 months post-injury appears to have some association to CSF cytokine levels (37–40). Elevated CSF IL-6, IL-8, and TNF seem to have the strongest associations with depression, apathy, and disinhibition at 6–12 months. Third, analysis of the impact of CSF cytokine levels on neurophysiologic measures is limited, with only five studies documenting such data (36, 48, 49, 51, 52). The strongest relationship identified here was the link between elevated levels of various cytokines, such as IL-6 or IL-1b, and elevated ICP (36, 48, 52). Further work is required before robust conclusions can be drawn in this area. Fourth, none of the studies explored the link between CSF cytokine measures and tissue outcome, as assessed by follow-up neuroimaging. Fifth, despite the “positive” associations found in the previously described papers, 11 manuscripts found no relationship between CSF cytokines and patient outcome (53–55, 58–65). The patient numbers in the individual studies, which reported no associations was much smaller than that in the studies describing a positive association between CSF cytokines and patient outcome (mean of 28 vs. 41 patients/study, respectively), making lack of power a possible cause of a negative result. Further patients in the “nil association” studies represented an overall smaller sample, totaling 376 patients vs. 948 patients in the “positive association” studies. Finally, complication reporting within the CSF cytokine studies was absent. Selective reporting bias here is a major concern.

### Limitations

Despite the interesting results of these two systematic reviews, there are significant study limitations, which need to be highlighted. Limitations with each separate review can be found within the subsections to follow.

#### CMD Cytokine Review

First, there were a small number of heterogenous studies found for the CMD review, with some manuscripts reporting on the same patient populations based on banked CMD samples. Most of these studies had patient cohort with unspecified heterogeneous patterns of injury in the setting of sTBI. The exceptions were the studies describing “diffuse” TBI patients only. These drawbacks limit the generalizability of the results to all patients with sTBI. Second, the ICU and surgical therapies received by these patients during CMD sample collection/processing was quite heterogeneous and poorly reported, and could have driven substantial variation in CMD cytokine measures. Third, there were variations in CMD catheter location between studies. This could impact the CMD cytokine measures obtained and the described relationships. Fourth, complications association with CMD monitoring was seldom reported. We believe there is significant selective harms reporting. Finally, given the studies and results identified for the CMD review, there is likely a large publication bias, favoring only studies with positive results.

#### CSF Cytokine Review

First, there were many quite heterogeneous studies identified in the CSF cytokine review. The included papers varied by study design, number of patients, patient inclusion criteria, ICU-based therapies offered/provided to patients, blinding during outcome assessment, and primary outcome of the studies. Information regarding the relationship between CSF cytokine measures and patient outcome was often buried within the text, and often not an explicit target for the study. Furthermore, selective outcome reporting with regards to individual CSF cytokine measures and their association to patient outcome was present in many studies. Thus, the conclusions that can be drawn from these studies and the strength of associations between CSF cytokines with patient outcome/neurophysiologic outcome are limited. Second, selective outcome reporting was an issue in many studies with preference to reporting significant association(s) only, making no reference to other CSF measures and the results of statistical analysis. Third, complication reporting was concerning within the literature identified (as mentioned above). Significant underreporting is suspected, with selective harms reporting the likely cause. Fourth, given all the above limitations and heterogeneity issues, a meta-analysis was not performed. Finally, though majority of studies report a positive association between cytokine levels and outcome, given that this is an emerging area of research, it is important to consider whether this might represent a publication bias toward positive studies.

#### Correlation with Clinical Parameters

Several studies attempt to correlate a specific mediator concentration with outcome. As these mediators are known to act in complex cascades and show a high degree of statistical collinearity, simple inferences cannot be made about the role of a given mediator in causing a particular outcome or relating to a clinical parameter such as ICP. As these mediators are induced by the initial traumatic insult, they are all confounded by severity of injury: it is, therefore, not surprising that a high concentration of cytokine relates to a worsened clinical parameter. Furthermore, the timing of monitoring in relation to the time of injury is not consistently reported. Several mediators, such as IL6, can have differing biological effects depending on the milieu in which they are produced (68). Finally, many mediators are known to act in concert and regulate the same downstream pathways (e.g., IL1b and IL1ra) such that measuring a mediator in isolation does not reflect its true biological role, which is time and milieu dependent.

### Future Directions

Given the significant heterogeneity in both study design, patient injury patterns, ICU/surgical treatments, and CMD/CSF cytokine measures identified within both systematic reviews, there is substantial room for more investigation into this emerging area of the literature in sTBI.

Although it is tempting to simply suggest that larger studies are done to overcome the heterogeneity in injury patterns following TBI, there are significant limitations to this approach. There are an ever-expanding list of mediators available for analysis over multiple time points in a range of biological fluids and without a robust understanding of the interaction between these mediators, it is unlikely that a meaningful pattern will emerge through brute force of numbers. More refined approaches that explore within patient comparisons with multiple sites of monitoring (69), interventional studies in which specific modulation of a biological pathway (16), and more sophisticated multivariate statistical methods (14).

Some studies have attempted to relate intensive care parameters such as ICP to the cytokine and chemokine response to TBI (26). This is not a simple relationship as the time frame over which cytokines and chemokines are produced occur over several days and weeks, rather than over the minutes and hours. There is insufficient evidence to stipulate, which intensive care interventions should be applied during monitoring of inflammatory mediators; however, it is important for individual studies to report their intensive care protocols and interventions.

As CMD is necessarily focal in nature, strict reporting of the method of localization is required and ideally 2 catheter studies, 1 in peri-lesional tissue and 1 in healthy tissue provides the most informative data (quote consensus paper).

When multiple mediators are measured, multivariate statistical methods must be employed, such as multivariate projection methods in order to model the potential interactions (14, 70).

This could potentially identify cytokine patterns of coexpression in CMD and CSF, highlighting target for future studies and therapeutic targets.

One deficit in the current CMD literature is complication reporting. In part, this relates to the difficulty in apportioning complications to CMD catheter insertion specifically. As patients will have invasive monitoring for ICP monitoring and brain tissue oxygenation in any circumstance for directing clinical therapy, the additional risk of inserting CMD through an existing cranial access device is small and difficult to quantify. Nevertheless, transparency dictates that complications are reported. Standardization of the methodologies employed allows multicenter prospective evaluation of cytokines within CDM and CSF and is necessary to improve patient recruitment and aid with spreading the substantial cost of cytokine analysis among centers. Without this collaboration, the limitations with single center recruitment and costs of cytokine processing in CMD and CSF limits the ability to combine datasets across units and studies. This would allow easier compilation of data sets and may add clarity to the associations highlighted within this manuscript. Finally, a consideration of the methodological factors that determine microdialysis catheter efficiency, including choice of perfusion fluid, catheter membrane, and pump flow rate all have an impact on the result obtained.

## Conclusion

The evaluation of CMD and CSF cytokines is an emerging area of the literature in sTBI. The two scoping systematic reviews have demonstrated a limited literature available on CMD cytokine measurement in sTBI, with some preliminary data supporting feasibility of measurement and associations between cytokines and patient outcome. Second, a number CSF cytokine levels may be associated with patient outcome at 6–12 months, including IL-1b, IL-1ra, IL-6, IL-8, IL-10, and TNF. Third, there is little to no literature to date in support of an association between CSF cytokines and neurophysiologic or tissue outcomes. Ultimately, the aim of CMD monitoring of inflammatory mediators is to reveal the underlying pathophysiology of TBI rather than as a clinical tool.

## Author Contributions

FZ was involved in project conception, design, systematic review searching, data extraction/tabulation, data interpretation, manuscript composition, and editing. ET was involved with data extraction/tabulation, manuscript composition, and editing. MC was involved in manuscript composition and editing. PH was involved in design, data interpretation, and manuscript editing. DM and AH was involved in design, data interpretation, manuscript writing, and editing.

## Conflict of Interest Statement

FZ has received salary support for dedicated research time, during which this project was partially completed. Such salary support came from: the Cambridge Commonwealth Trust Scholarship, the Royal College of Surgeons of Canada—Harry S. Morton Traveling Fellowship in Surgery, the University of Manitoba Clinician Investigator Program, R. Samuel McLaughlin Research and Education Award, the Manitoba Medical Service Foundation, and the University of Manitoba—Faculty of Medicine Dean’s Fellowship Fund. ET has received funding support from Swedish Society of Medicine (Grant no. SLS-587221). MC has financial interest in a part of licensing fee for ICM+ software (Cambridge Enterprise Ltd., UK). Unpaid co-director of Technicam Ltd.–producer of Cranial Access Device used for CMD insertion. PH is the director of Technicam manufacturer of the Technicam Cranial Access Device. DM has consultancy agreements and/or research collaborations with GlaxoSmithKline Ltd.; Ornim Medical; Shire Medical Ltd.; Calico Inc.; Pfizer Ltd.; Pressura Ltd.; Glide Pharma Ltd.; and NeuroTraumaSciences LLC.

The reviewer, FC, and handling editor declared their shared affiliation, and the handling editor states that the process met the standards of a fair and objective review.
