# Supplementary material for: Cerebrospinal Fluid and Microdialysis Cytokines in Severe Traumatic Brain Injury: A Scoping Systematic Review
Source: Front Neurol. 2017 Jul 10;8:331. doi: 10.3389/fneur.2017.00331 (PMC5502380; doi:10.3389/fneur.2017.00331)
Supplement: Supplementary file 1 [file data_sheet_1.doc]

**Appendix A – TBI CMD Cytokine Systematic Review - MEDLINE Search Strategy**

1. traumatic brain injury.mp. [mp=ti, ab, hw, tn, ot, dm, mf, dv, kw, fs, bt, id, cc, nm, kf, px, rx, an, eu, pm, ui]

2. traumatic brain injury.tw.

3. TBI.mp.

4. TBI.tw.

5. brain injury.mp.

6. brain injury.tw.

7. head injury.mp.

8. head injury.tw.

9. head trauma.mp.

10. head trauma.tw.

11. cerebral trauma.mp.

12. cerebral trauma.tw.

13. brain trauma.mp.

14. brain trauma.tw.

15. cerebral injury.mp.

16. cerebral injury.tw.

17. craniocerebral trauma.mp.

18. craniocerebral trauma.tw.

19. cranial trauma.mp.

20. cranial trauma.tw.

21. cranial injury.mp.

22. cranial injury.tw.

23. cytokine.mp.

24. cytokine.tw.

25. chemokine.mp.

26. chemokine.tw.

27. beta-thromboglobulin.mp.

28. beta-thromboglobulin.tw.

29. chemokine c.mp.

30. chemokine c.tw.

31. chemokine cc.mp.

32. chemokine cc.tw.

33. chemokine cx3c.mp.

34. chemokine cx3c.tw.

35. chemokine cxc.mp.

36. chemokine cxc.tw.

37. inflammatory proteins.mp.

38. inflammatory proteins.tw.

39. factor 15.mp.

40. factor 15.tw.

41. hematopoietic cell growth factor 15.mp.

42. hematopoietic cell growth factor.mp.

43. hematopoietic growth factor.tw.

44. colony stimulating factor.mp.

45. colony stimulating factor.tw.

46. stem cell factor.mp.

47. stem cell factor.tw.

48. hepatocyte growth factor.mp.

49. hepatocyte growth factor.tw.

50. interferon.mp.

51. interferon.tw.

52. interferon gamma.mp.

53. interferon gamma.tw.

54. interferon type 1.mp.

55. interferon type 1.tw.

56. interleukin 1 receptor.mp.

57. interleukin 1 receptor.tw.

58. interleukin.mp.

59. interleukin.tw.

60. interleukin 1.mp.

61. interleukin 1.tw.

62. interleukin 2.mp.

63. interleukin 2.tw.

64. interleukin 3.mp.

65. interleukin 3.tw.

66. interleukin 4.mp.

67. interleukin 4.tw.

68. interleukin 5.mp.

69. interleukin 5.tw.

70. interleukin 6.mp.

71. interleukin 6.tw.

72. interleukin 7.mp.

73. interleukin 7.tw.

74. interleukin 8.mp.

75. interleukin 8.tw.

76. interleukin 9.mp.

77. interleukin 9.tw.

78. interleukin 10.mp.

79. interleukin 10.tw.

80. interleukin 11.mp.

81. interleukin 11.tw.

82. interleukin 12.mp.

83. interleukin 12.tw.

84. interleukin 13.mp.

85. interleukin 13.tw.

86. interleukin 15.mp.

87. interleukin 15.tw.

88. interleukin 16.mp.

89. interleukin 16.tw.

90. interleukin 17.mp.

91. interleukin 17.tw.

92. interleukin 18.mp.

93. interleukin 18.tw.

94. interleukin 23.mp.

95. interleukin 23.tw.

96. interleukin 27.mp.

97. interleukin 27.tw.

98. interleukin 33.mp.

99. interleukin 33.tw.

100. Leukemia inhibitory factor.mp.

101. leukemia inhibitory factor.tw.

102. macrophage activating factor.mp.

103. macrophage activating factor.tw.

104. macrophage migrating inhibitory factor.mp.

105. macrophage migating inhibtory factor.tw.

106. immunologic transfer factor.mp.

107. immunologic transfer factor.tw.

108. lymphotoxin alpha.mp.

109. lymphotoxin alpha.tw.

110. monokine.mp.

111. monokine.tw.

112. tumor necrosis factor alpha.mp.

113. tumor necrosis factor alpha.tw.

114. oncostatin m.mp.

115. oncostatin m.tw.

116. osteopontin.mp.

117. osteopontin.tw.

118. transforming growth factor beta.mp.

119. transforming growth factor beta.tw.

120. transforming growth factor beta 1.mp.

121. transforming growth factor beta 1.tw.

122. transforming growth factor beta 2.mp.

123. transforming growth factor beta 2.tw.

124. transforming growth factor beta 3.mp.

125. transforming growth factor beta 3.tw.

126. tumor necrosis factor.mp.

127. tumor necrosis factor.tw.

128. 1 or 2 or 3 or 4 or 5 or 6 or 7 or 8 or 9 or 10 or 11 or 12 or 13 or 14 or 15 or 16 or 17 or 18 or 19 or 20 or 21 or 22

129. 23 or 24 or 25 or 26 or 27 or 28 or 29 or 30 or 31 or 32 or 33 or 34 or 35 or 36 or 37 or 38 or 39 or 40 or 41 or 42 or 43 or 44 or 45 or 46 or 47 or 48 or 49 or 50 or 51 or 52 or 53 or 54 or 55 or 56 or 57 or 58 or 59 or 60 or 61 or 62 or 63 or 64 or 65 or 66 or 67 or 68 or 69 or 70 or 71 or 72 or 73 or 74 or 75 or 76 or 77 or 78 or 79 or 80 or 81 or 82 or 83 or 84 or 85 or 86 or 87 or 88 or 89 or 90 or 91 or 92 or 93 or 94 or 95 or 96 or 97 or 98 or 99 or 100 or 101 or 102 or 103 or 104 or 105 or 106 or 107 or 108 or 109 or 110 or 111 or 112 or 113 or 114 or 115 or 116 or 117 or 118 or 119 or 120 or 121 or 122 or 123 or 124 or 125 or 126 or 127

130. 128 and 129

131. cerebral microdialysis.mp.

132. cerebral microdialysis.tw.

133. microdialysis.mp.

134. microdialysis.tw.

135. dialysis.mp.

136. dialysis.tw.

137. MDialysis.mp.

138. MDialysis.tw.

139. md probe.mp.

140. md probe.tw.

141. microdialysate.mp.

142. microdialysate.tw.

143. dialysate.mp.

144. dialysate.tw.

145. 131 or 132 or 133 or 134 or 135 or 136 or 137 or 138 or 139 or 140 or 141 or 142 or 143 or 144

146. 130 and 145
